# Visualization of PAX7 protein dynamics in muscle satellite cells in a YFP knock-in-mouse line

**DOI:** 10.1186/s13395-018-0174-x

**Published:** 2018-08-24

**Authors:** Yasuo Kitajima, Yusuke Ono

**Affiliations:** 1 Musculoskeletal Molecular Biology Research Group, Basic and Translational Research Center for Hard Tissue Disease, Nagasaki University Graduate School of Biomedical Sciences, 1-7-1 Sakamoto, Nagasaki, 852-8588 Japan; 2 Japan Society for the Promotion of Science, 5-3-1 Kojimachi, Chiyoda-ku, Tokyo, 102-0083 Japan; 3 Agency for Medical Research and Development (AMED), 1-7-1 Otemachi, Chiyodaku, Tokyo, 100-0004 Japan

**Keywords:** Pax7, Knock-in mouse, YFP, Myogenesis, Muscle regeneration

## Abstract

**Background:**

Satellite cells are residential muscle stem cells that express a paired box protein, PAX7.

**Results:**

Here, we report a knock-in mouse line expressing a PAX7-enhanced yellow fluorescent protein (YFP) fusion protein that enables visualization of PAX7 protein dynamics in living satellite cells through YFP fluorescence. The YFP fluorescence signals in Pax7-YFP knock-in mice clearly recapitulated the endogenous expression of PAX7 protein in satellite cells. YFP+ satellite cells were efficiently isolated from muscle tissues by fluorescence-activated cell sorting. Homozygous Pax7-YFP knock-in mice (Pax7^YFP/YFP^) were viable, grew and regenerated muscle normally, and Pax7^YFP/YFP^ mouse-derived satellite cells proliferated, differentiated, and self-renewed as efficiently as those from wild-type (Pax7^+/+^) mice.

**Conclusions:**

Taken together, our Pax7-YFP mouse line is a useful tool to aid the development of stem-cell-based therapies for muscle diseases.

**Electronic supplementary material:**

The online version of this article (10.1186/s13395-018-0174-x) contains supplementary material, which is available to authorized users.

## Background

Skeletal muscle retains a remarkable capacity to regenerate. This regenerative capacity depends on residential stem cells called muscle satellite cells that provide myonuclei not only for regeneration in the adult but also for postnatal muscle growth [1–6]. Satellite cells are located between the basal lamina and the plasmalemma of myofibers and are mitotically quiescent in healthy adult muscle [7]. Transplantation analysis shows that satellite cells possess potent myogenic and self-renewal abilities to reconstitute host muscle in vivo [8–13]. In contrast, depletion of the satellite cell population by inducing expression of diphtheria toxin fragment A [14–16] or a failure of satellite cell function [17] results in severe loss of regenerated muscle. Together, these data provide direct evidence that the satellite cell population is indispensable for regeneration in adult muscle.

Satellite cells are normally quiescent but become activated in response to stimulation including traumatic muscle injury. After activation, they enter the cell cycle and give rise to proliferative satellite-cell progeny, called myoblasts. Myoblasts then undergo myogenic differentiation and either fuse with existing myofibers or form new myofibers by producing myonuclei. Meanwhile, a minority of the population return to a quiescent state to self-renew, maintaining the stem-cell pool [8, 12, 18–21].

The paired box protein, PAX7, is a transcription factor that is uniformly expressed in quiescent to proliferative state satellite cells. However, it is downregulated during myogenic differentiation [18, 20]. PAX7 plays important roles in satellite cell survival, specification, proliferation, and differentiation [19, 22–28]. Mice lacking the *Pax7* gene are viable until 2–3 weeks after birth with a marked reduction in body-size [23, 27]. *Pax7*-null mice exhibit a progressive loss of satellite cells in muscle during growth, because of a decrease in proliferation and precocious myogenesis, leading to a significant decrease in myonuclear-numbers and myofiber-diameters [23, 25, 27, 29]. More recently, satellite-cell-specific inactivation of *Pax7* induced by tamoxifen injection in mice resulted in a reduced satellite cell number, a proliferative defect, and precocious myogenic differentiation, resulting in a severe impairment in muscle regeneration [30–32]. Together, these findings illustrate that PAX7 expressed in satellite cells is essential not only during the juvenile period to give rise to progeny but also during muscle regeneration in adults [30, 31, 33].

Here, we generated a mouse line carrying the PAX7 protein fused with enhanced yellow fluorescent protein (YFP) that enables indirect visualization of endogenous PAX7 protein dynamics in living satellite cells. YFP+ satellite cells could be efficiently isolated by fluorescence-activated cell sorting (FACS) without antibody staining and were transplantable, similarly to cells isolated from transgenic Pax7-ZsGreen, Pax7-nGFP, and Pax7-GFP reporter mice that have recently been reported [34–36]. Importantly, the YFP-tag does not interfere with the function of the endogenous PAX7 protein because Pax7^*YFP/YFP*^ homozygous mice are born, grow, and regenerate muscle normally, and Pax7^YFP/YFP^ mouse-derived satellite cells undergo proliferation, myogenic differentiation, and self-renewal, similar to wild-type satellite cells. Although the fluorescence intensity of YFP-tagged PAX7 protein is lower than other reporter lines, our Pax7-YFP mouse line allows not only further characterization of satellite cell dynamics but also the visualization and biochemical analysis of endogenous PAX7 protein dynamics. Thus, our newly established knock-in mouse line will be an additional useful tool for the researchers in the field of muscle biology and facilitate the development of stem-cell-based therapies for muscle diseases.

## Methods

### Antibodies and reagents

Antibodies and reagents were obtained from the following sources. PE-conjugated anti-CD31, anti-CD45, and anti-Sca-1 and APC-conjugated anti-Vcam1 antibodies were obtained from BioLegend (San Diego, CA, USA). Rabbit or mouse anti-GFP antibodies cross-reacting with YFP were obtained from Thermo Fisher Scientific (Carlsbad, CA, USA) or EMD Millipore. Mouse anti-PAX7 and mouse anti-myosin heavy chain (MF20, MAB4470) antibodies were purchased from R&D Systems (Minneapolis, MN, USA). Rabbit anti-MyoD antibody was from Santa Cruz Biotechnology (Santa Cruz, CA, USA). Rabbit anti-Laminin antibody was obtained from Sigma (Sigma-Aldrich, St. Louis, MO). Rat anti-Laminin α2 antibody was obtained from Enzo (Enzo Life Sciences, NY). Rabbit anti-Dystrophin antibody was obtained from Abcam (Cambridge, MA, USA). Rat anti-Ki67 antibody and DAKO Protein Block were obtained from DAKO (Tokyo, Japan). Alexa Fluor-conjugated secondary antibodies were purchased from Thermo Fisher Scientific. M.O.M. kit and mounting medium containing 4,6-diamidino-2-phenylindole (DAPI) for nuclear staining was obtained from Vector Laboratories (Burlingame, CA, USA).

### Generation of Pax7-YFP knock-in mouse line

**Fig. 1:**
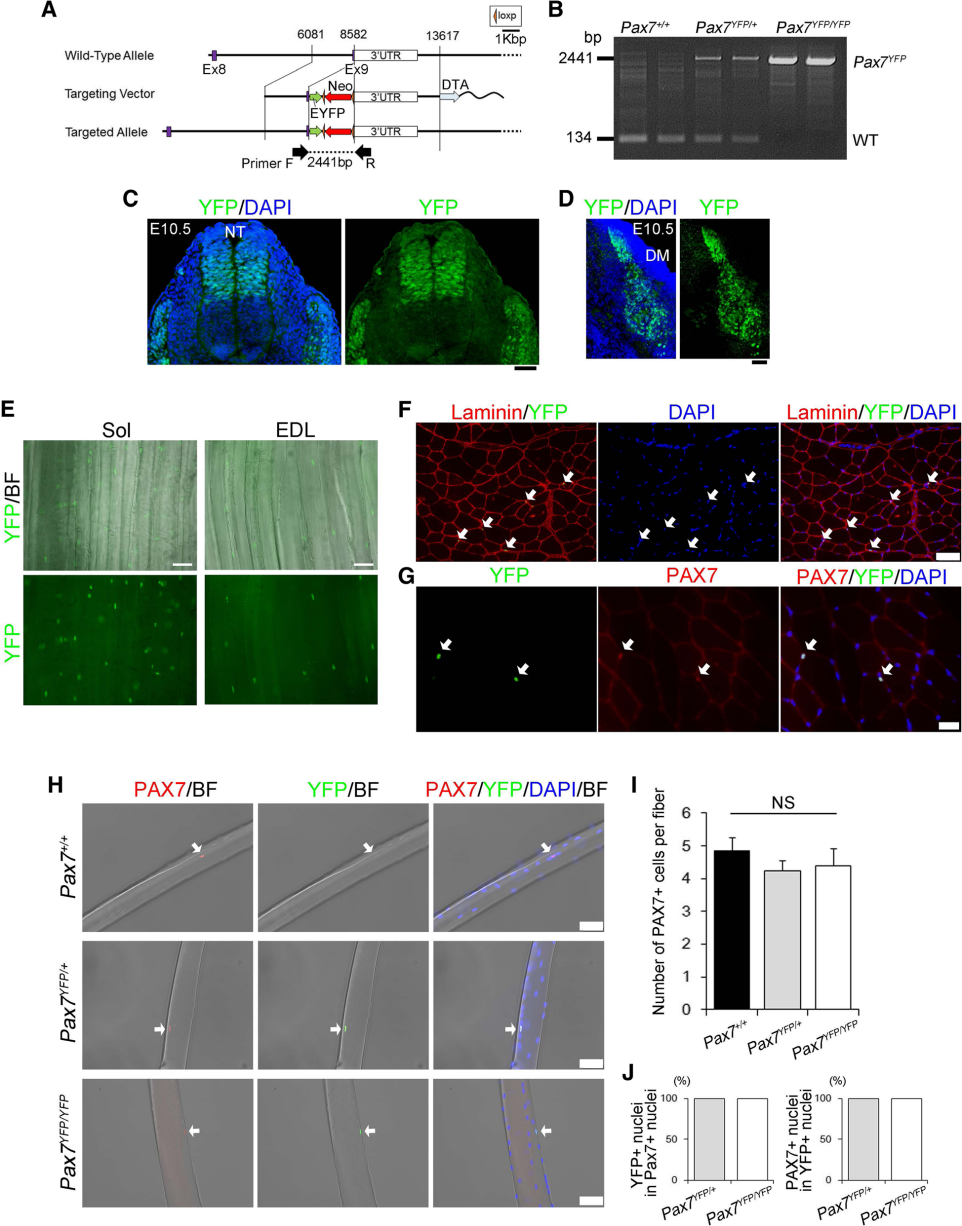
Generation of Pax7-YFP knock-in mice. **a** Schematic diagrams showing the knock-in construct and knock-in allele. A targeting vector for generating a Pax7-YFP knock-in mouse line was constructed by inserting an EYFP sequence downstream of exon 9 of the *Pax7* gene locus. **b** Genotype of Pax7-YFP knock-in mice was verified by a two-primer PCR strategy (wild-type = 134 bp, Pax7-EYFP = 2441 bp). Pax7^+/+^, wild-type; Pax7^YFP/+^, heterozygous Pax7^YFP/+^; Pax7^YFP/YFP^, homozygous Pax7^YFP/YFP^. **c** Immunohistochemistry of YFP in the neural tube (NT) and somite region of a Pax7^YFP/YFP^ mouse embryo at E10.5, scale bar 50 μm. **d** YFP fluorescence signals in the dermomyotome (DM) region of a Pax7^YFP/YFP^ mouse embryo at E10.5, scale bar 50 μm. **e** Representative fluorescence images of YFP+ cells in soleus (Sol) and extensor digitorum longus (EDL) muscle tissues freshly isolated from Pax7^YFP/YFP^ mice, scale bar 50 μm. **f** Immunohistochemistry of YFP and laminin α2 in cryosections of TA muscle from adult Pax7^YFP/YFP^ mice. Arrows indicate YFP^+^ cells, scale bar 50 μm. **g** Immunohistochemistry showed that YFP+ cells were co-localized with PAX7+ satellite cells in transverse sections of TA muscle in Pax7^YFP/YFP^ mice. Arrows indicate YFP + PAX7+ satellite cells, scale bar 20 μm. **h**-**j** Immunostaining showed that YFP + PAX7+ satellite cells associated with individual myofibers freshly isolated from EDL muscle in Pax7^+/+^, Pax7^YFP/+^, and Pax7^YFP/YFP^ mice. Arrows indicate PAX7+ satellite cells. **h** The number of satellite cells per myofiber was quantified in (**i**). YFP+ cells totally overlapped PAX7+ satellite cells (**j**) (*n* = 3–5, > 20 myofibers per animal were counted), scale bar 20 μm. DAPI was used to visualize nuclear staining. Data represent means ± s.e.m. (one-way ANOVA followed by the Bonferroni post hoc test). NS non-significant

The Experimental Animal Care and Use Committee of Nagasaki University approved all animal experimentation used in this study (ref. no. 1203190970). The BRUCE-4 ES cell line (C57/BL6J) was used to generate the Pax7-YFP knock-in mouse line. A targeting vector was generated to modify the *Pax7* gene by inserting an EYFP sequence downstream of the terminal exon 9 of *Pax7* (Fig. 1a). To express a Pax7-YFP fusion protein, the only stop codon of exon 9 was deleted. Briefly, an EYFP-loxP flanked Neo cassette was replaced with the terminal exon 9 of *Pax7* to construct the Pax7-YFP knock-in vector. The Neo cassette was not removed. The genotype of the transgenic Pax7-YFP knock-in (KI) mice was verified by PCR using the following primer pair (Fig. 1b); forward primer 5′-AGCGCCGTATGAAGCTTGGG-3′, reverse primer 5′-AAGGGGACTGAGGTGAGGAGA-3′, (wild-type = 134 bp, Pax7-YFP = 2441 bp). Male mice between 7 and 14 weeks of age were used in all experiments.

### Muscle injury

Cardiotoxin (CTX, Sigma-Aldrich) was prepared by dissolving a freshly opened tube in 0.9% NaCl at 10 μM. To induce muscle injury, 100 μl of 10 μM CTX was injected intramuscularly into the tibialis anterior (TA) muscle of anesthetized mice. Regenerating muscles were isolated at 3, 7, and 14 days after CTX injection. TA muscles were frozen in either 2-methylbutane cooled with liquid nitrogen or liquid nitrogen for histological analysis or RNA isolation, respectively, and stored at − 80 °C. Transverse muscle sections were cut using a cryostat.

### Transplantation

Dystrophin-null mdx mutant mice, a mouse model for Duchenne muscular dystrophy (DMD), were used as a recipient animal. Regeneration of TA muscle in mdx mice was induced by intramuscular injection of 100 μl CTX 1 day before transplantation. Pax7^YFP/YFP^ mice were used as a donor animal. YFP^+^ satellite cells (5 × 10^4^ cells) were freshly isolated from Pax7^YFP/YFP^ mice by FACS and were transplanted into CTX-pretreated TA muscles of mdx mice. TA muscles were harvested 2 weeks after transplantation and stored at − 80 °C.

### FACS analysis

Hindlimb muscles were collected and excess fat, connective tissue, and tendons removed. Mononuclear cells from hindlimb muscles were prepared using 0.2% collagenase type II (Worthington Biochemical) as previously described [37]. Mononuclear cells were stained with PE-conjugated anti-CD31, anti-CD45, and anti-Sca-1 and APC-conjugated anti-Vcam1 antibodies on ice for 30 min and resuspended in PBS containing 2% FBS. Cell sorting was performed using a FACS Aria II flow cytometer (BD Immunocytometry Systems). Debris and dead cells were excluded by forward scatter, side scatter, and PI gating. Data were collected using FACS Diva software (BD Biosciences).

### Myofiber and satellite cell isolation and culture

Individual myofibers were isolated from the extensor digitorum longus (EDL) muscles, as described previously [38]. In brief, EDL muscles were digested using 0.2% type I collagenase (Worthington Biochemical) in DMEM for 90 min at 37 °C under 5% CO_2_. For immunocytochemical analysis, EDL myofibers were immediately fixed using 4% paraformaldehyde (PFA). Satellite cells were obtained from isolated myofibers and cultured in growth medium (GM; DMEM supplemented with 30% fetal bovine serum, 1% chicken-embryo extract, 10 ng/ml basic fibroblast growth factor, and 1% penicillin-streptomycin) at 37 °C under 5% CO_2_ immediately after isolation. Myogenic differentiation was induced in differentiation medium (DM; DMEM supplemented with 5% horse serum and 1% penicillin-streptomycin) at 37 °C under 5% CO_2_.

### Immunostaining

Immunocytochemistry of satellite cells and isolated single myofibers was performed as described previously [38]. Samples were fixed with 4% PFA, blocked/permeabilized with phosphate-buffered saline containing 0.3% Triton X-100 and 5% goat serum for 20 min at room temperature, and incubated with primary antibodies at 4 °C overnight. For immunohistochemistry, cryosections of TA muscle tissues and E10.5 embryos were fixed with 4% PFA, blocked with 5% goat serum or the M.O.M kit (Vector Laboratories) for 30 min at room temperature and incubated with primary antibodies at 4 °C overnight. All immunostained samples were visualized using appropriate species-specific Alexa Fluor fluorescence-conjugated secondary antibodies. Samples were viewed on an Olympus IX83 microscope (Olympus, Tokyo, Japan) or on a Cell Insight CX5 (Thermo Fisher Scientific). Digital images were acquired and quantified with a DP80 camera using cellSens software (Olympus) or with a Photometrics X1 camera using HCS Studio software (Thermo Fisher Scientific). Images were optimized globally and assembled into figures using Adobe Photoshop. Immunostaining for laminin to measure cross-sectional area (CSA) of centrally nucleated regenerating myofibers was performed, and CSA was quantified using cellSens software (Olympus). For EdU detection, the Click chemical reaction was performed after primary and secondary staining according to the manufacturer’s instructions using a Click-iT EdU Imaging Kit (Thermo Fisher Scientific).

### Quantitative revere transcription-PCR (Q-PCR)

Total RNA was isolated using an RNAeasy Kit (Qiagen, Hilden, Germany). For real-time PCR, first-strand cDNA was synthesized using oligo-dT primers (Toyobo). The expression levels of selected genes were analyzed using a CFX96 real-time PCR detection system (Bio-Rad, Tokyo, Japan) according to the manufacturer’s instructions. Primer sequences were listed in Additional file 1.

### Statistical analysis

Statistical analyses were performed with SPSS software (IBM) to determine significant differences from a two-tailed distribution using the paired or unpaired Student’s *t* test. In comparisons of more than two groups, non-repeated measures analysis of variance (ANOVA) followed by the Bonferroni post hoc test was used. *P* values are indicated on each figure as < 0.05 (*), < 0.01 (**), and < 0.001 (***). All error bars are indicated as means ± s.d. or s.e.m. NS indicates statistically non-significant.

## Results

### Generation of a PAX7-YFP mouse line

To generate a mouse line expressing a PAX7 and YFP fusion protein (PAX7-YFP), we constructed a targeting vector with the enhanced YFP gene inserted into the end of exon 9 of the endogenous *Pax7* locus with the endogenous *Pax7* stop codon deleted (Fig. 1a, b). The Pax7 gene is highly expressed in the craniofacial region and somites [34, 39, 40]. Consistent with the expression pattern of PAX7 in mouse embryos [22, 24], immunohistochemistry showed that YFP-positive cells were localized in the neural tube and dermomyotome in homozygous (Pax7^YFP/YFP^) E10.5 embryos (Fig. 1c, d). Although native YFP fluorescence signal recapitulated the endogenous expression pattern of PAX7 in whole homozygous embryos, its fluorescence intensity was not strong enough to detect the defined PAX7-expressing areas in the somites under the fluorescence stereo microscopy (data not shown).

To determine whether the YFP fluorescence signal recapitulated the localization of endogenous PAX7 protein in adult muscle, freshly isolated soleus (Sol), extensor digitorum longus (EDL), and tibialis anterior (TA) muscles were obtained from 12-week-old Pax7^YFP/YFP^ mice immediately after sacrifice. YFP-positive signals were detected between individual myofibers, which is the expected localization of satellite cells (Fig. 1e). In general, the Sol muscle contains more satellite cells than other limb muscles such as TA and EDL muscles [8, 10], indicating that the YFP-positive signals accurately represent the number of satellite cells per myofiber in mouse muscles. Immunohistochemical analysis showed co-localization between YFP^+^ nuclei and PAX7^+^ nuclei in cross-sections of TA muscle (Fig. 1f, g). Importantly, all YFP^+^ nuclei corresponded to PAX7^+^ nuclei of satellite cells located between myofibers of the EDL muscle in both Pax7^YFP/+^ and Pax7^YFP/YFP^ mice (Fig. 1h–j). These data indicate that expression of the PAX7-YFP fusion protein faithfully recapitulated the expression pattern of PAX7 protein, allowing us to indirectly and accurately detect the dynamics of endogenous PAX7 expression via YFP detection.

### Isolation of the satellite cell population from Pax7-YFP knock-in mice by FACS

**Fig. 2:**
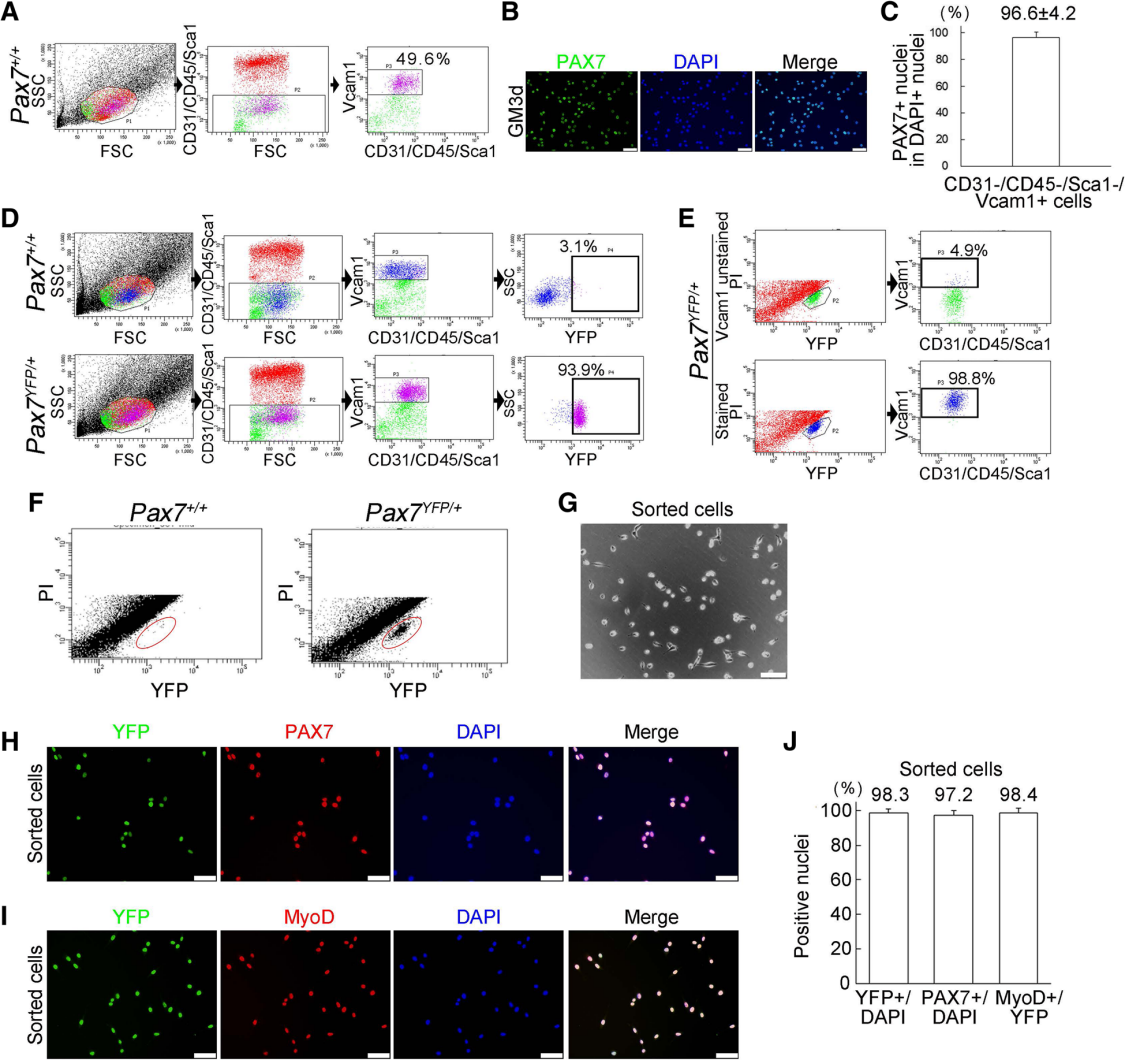
Satellite cell population is sortable from Pax7-YFP mice. **a**–**c** FACS profiles show a CD31^−^CD45^−^Sca1^−^Vcam1^+^ cell fraction of mononuclear cells isolated from limb muscles of wild-type mice (**a**). CD31^−^CD45^−^Sca1^−^Vcam1^+^ cells were sorted by FACS and stained with a PAX7 antibody (**b**) [quantified in (**c**)], scale bar 50 μm. Data represent the mean ± s.e.m. (*n* = 3). **d** A CD31^−^CD45^−^Sca1^−^Vcam1+ cell fraction was almost completely positive for YFP fluorescence. Top panel: FACS profiles of mononuclear cells derived from limb muscles of wild-type (Pax7^+/+^) mice. Bottom panel: FACS profiles of mononuclear cells derived from limb muscles of Pax7^YFP/+^ mice. **e** FACS plots demonstrated that YFP^+^ cells isolated from limb muscle of Pax7^YFP/+^ mice were positive for Vcam1 and negative for CD31, CD45, and Sca1. YFP^+^ cells were stained with (lower panel) or without (upper panel) a Vcam1 antibody. **f**–**j** A YFP^+^ cell population was isolated from limb muscles of Pax7^YFP/+^ mice by FACS without antibody staining. FACS profiles of mononuclear cells derived from Pax7^+/+^ or Pax7^YFP/+^ mice (**f**). The red circles indicate YFP^+^ cells. A representative image of isolated YFP^+^ cells cultured in growth medium (GM) for 3 days (**g**), scale bar 100 μm. YFP^+^ cells cultured in GM for 3 days as described in (**g**) were co-immunostained for YFP and PAX7 (**h**) or YFP and MyoD (**i**), scale bar 50 μm. Sorted YFP+ cells were quantified (**j**). Data represent the mean ± s.e.m. (*n* = 4)

We next validated whether YFP+ satellite cells in Pax7^YFP/+^ mice can be purified by FACS. For satellite-cell-sorting, a CD31^−^CD45^−^Sca1^−^Vcam1^+^ mononuclear fraction [41–43] was obtained from limb muscles of wild-type mice (Fig. 2a–c). FACS analysis showed that 98.8% of YFP+ cells sorted from Pax7^YFP/+^ mice by FACS overlapped with the CD31^−^CD45^−^Sca1^−^Vcam1^+^ mononuclear fraction (Fig. 2d, e), indicating that the satellite cell fraction could be isolated from Pax7^YFP/+^ mice with YFP fluorescence without antibody staining. Immunostaining also confirmed that a FACS-sorted YFP+ cell fraction contained 97.2% PAX7+ or 98.4% MyoD+ cells (Fig. 2f–j). Thus, our validation demonstrated that the satellite cell population could be prospectively and efficiently isolated from muscles of Pax7^YFP/+^ mice by FACS.

### YFP mirrors the dynamics of endogenous PAX7 in satellite cells

**Fig. 3:**
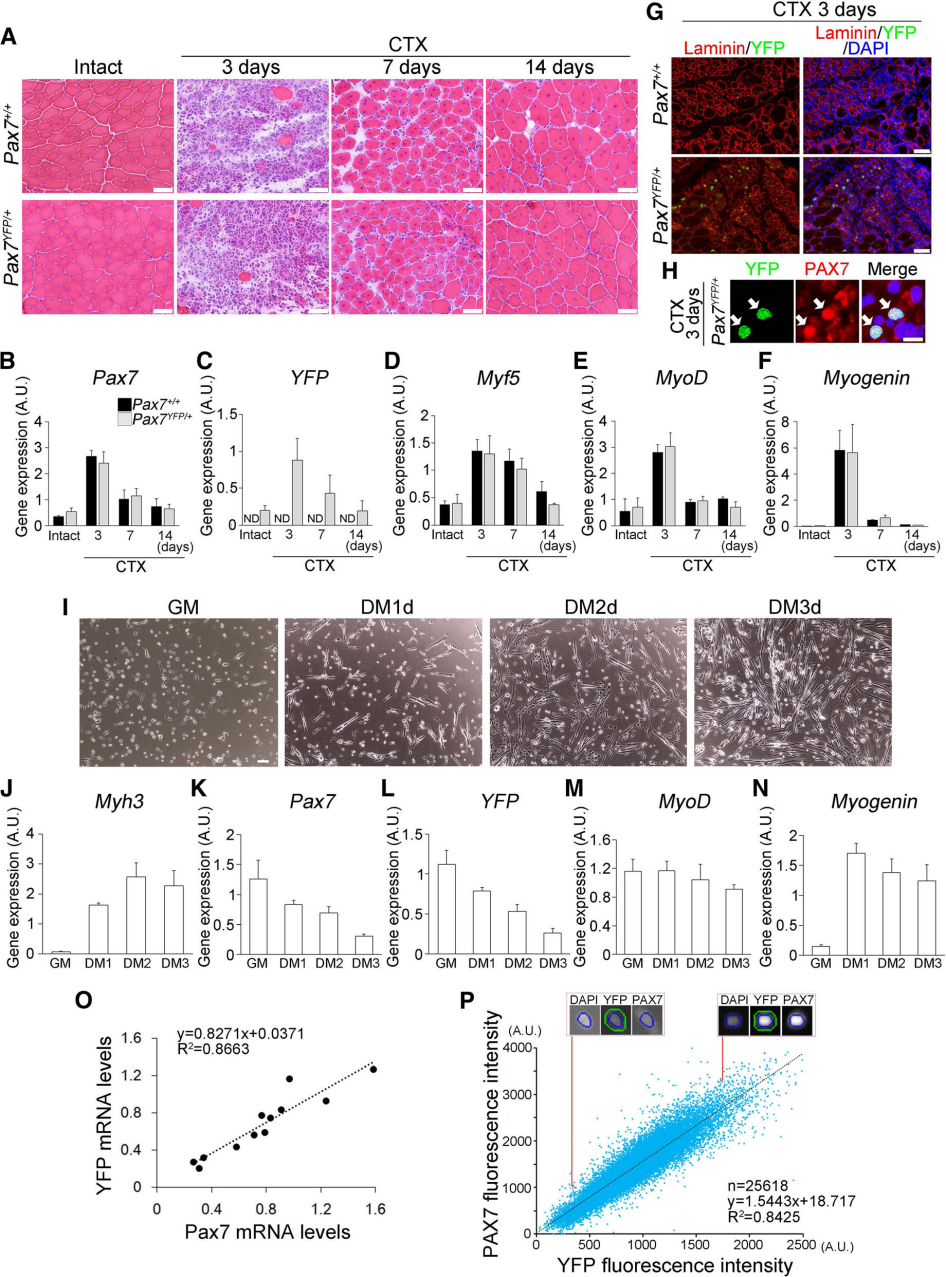
YFP expression levels mirror the endogenous dynamics of PAX7. **a**–**h** Regeneration of the TA muscle was induced by injection of cardiotoxin (CTX). H&E staining of cross-sections of TA muscle in Pax7^+/+^ and Pax7^YFP/+^ mice, 0, 3, 7, and 14 days following CTX injection, scale bar 50 μm (**a**). Q-PCR analysis of gene expression dynamics of myogenic regulatory factors (Pax7, Myf5, MyoD, and Myogenin) and YFP during muscle regeneration (**b**–**f**). Data represent means ± s.e.m. (ND, not detected; *n* = 3–5, per group). **g** Immunohistochemistry for YFP and Laminin α2 proteins in cross-sections of TA muscle isolated from Pax7^+/+^ or Pax7^YFP/+^ mice at day 3 after CTX injection, scale bar 50 μm. **h** Immunohistochemistry for YFP and PAX7 proteins in cross-sections of TA muscle isolated from Pax7^YFP/+^ mice at day 3 after CTX injection, scale bar 10 μm. **i**–**o** Satellite cells were isolated from limb muscles of Pax7^YFP/+^ mice and cultured in growth medium (GM). Myogenic differentiation was induced by differentiation medium (DM) for 3 days. Representative images are shown (**i**), scale bar 100 μm. **j**–**o** Q-PCR analysis shows a positive correlation between Pax7 and YFP mRNA levels during myogenic differentiation [quantified in (**o**) (*R*^2^ = 0.8663; *n* = 12)]. Data represent the mean ± s.d. (*n* = 3, each group). **p** Co-immunostaining revealed a positive correlation between YFP and PAX7 protein levels in satellite cells maintained under GM conditions. Fluorescence intensity was measured using the CellInsight CX5 Platform

In normal muscle regeneration, satellite cells are activated, proliferated, and then committed to myogenic differentiation to give rise to newly formed myofibers. To examine whether expression dynamics of the *Pax7-YFP* fusion gene mirrors endogenous *Pax7* gene expression during muscle regeneration, muscle injury was induced by intramuscular injection of CTX into the TA muscle. We compared the expression levels of the endogenous *Pax7* gene in regenerating muscles of wild-type mice with those of the *YFP* gene from Pax7^YFP/+^ mice. Expression patterns of *YFP* and *Pax7* genes were highly similar between Pax7^+/+^ and Pax7^YFP/+^ mice with peak expression at day 3 following CTX injection (Fig. 3a–f), when activated satellite cell progeny normally undergo population expansion [44]. Furthermore, we confirmed that YFP^+^ satellite cells in regenerative muscles were clearly detectable by immunohistochemistry (Fig. 3g, h).

Next, we examined whether the coincident expression of *YFP* and *Pax7* was also observed in plated satellite cells during myogenic progression. Satellite cells were isolated from limb muscles of Pax7^YFP/+^ mice by FACS and maintained in growth medium (GM) before induction of myogenic differentiation using differentiation medium (DM) for 3 days (Fig. 3i). Q-PCR analysis demonstrated a strong correlation between *Pax7* and *YFP* mRNA levels during myogenic progression (Fig. 3j–o). Moreover, co-immunostaining for PAX7 and YFP revealed almost identical fluorescence levels in plated satellite cells under GM conditions (Fig. 3p). Taken together, our data illustrate mirrored dynamics between PAX7 and YFP in satellite cells isolated from Pax7^YFP/+^ mice.

### Pax7-YFP homozygous mice grow and regenerate muscle normally

**Fig. 4:**
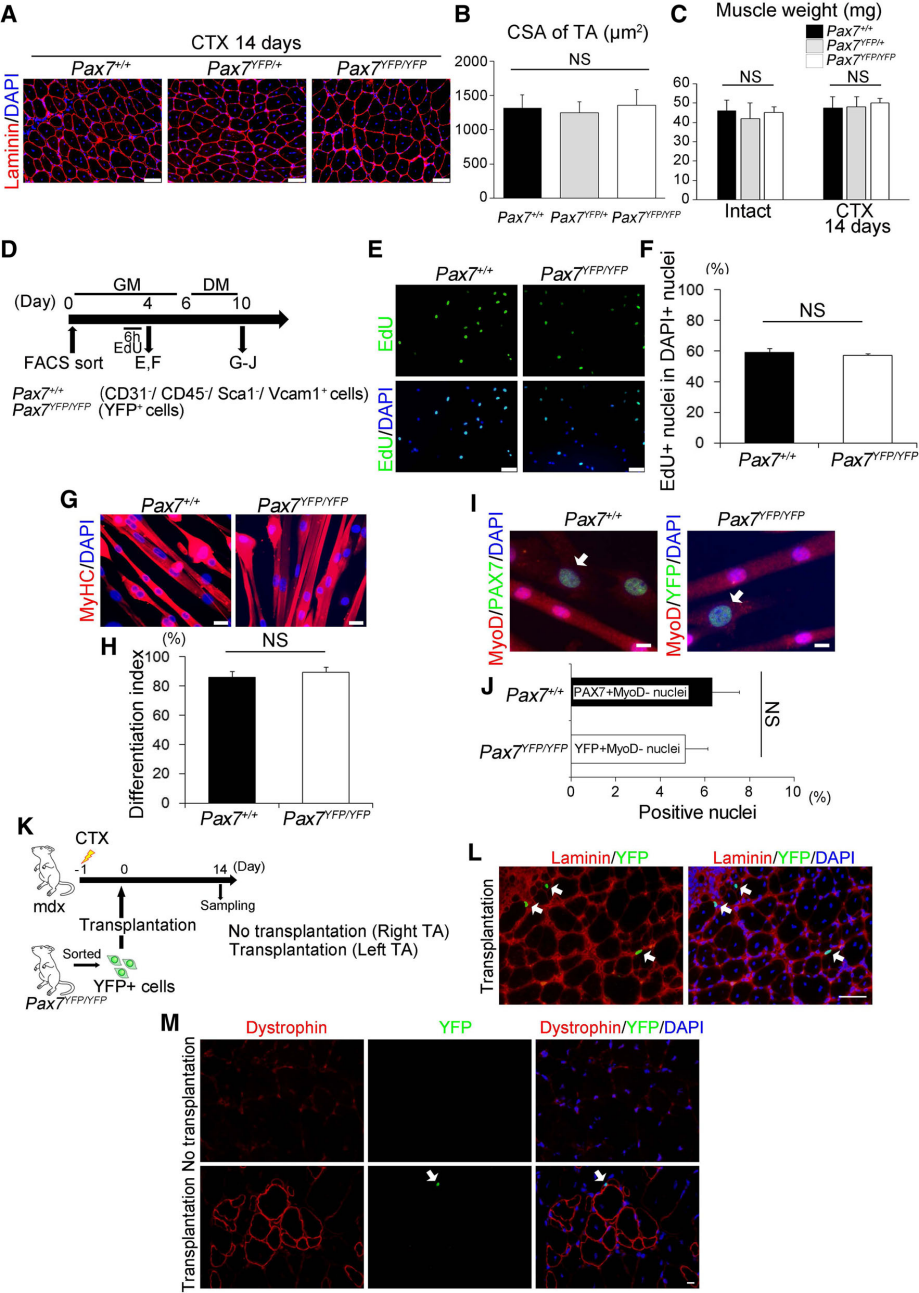
Homozygous Pax7-YFP mouse-derived satellite cells exhibit a wild-type stem cell function. **a**–**c** Regeneration was induced in the TA muscle of Pax7^+/+^, Pax7^YFP/+^, and Pax7^YFP/YFP^ mice by injection of CTX as shown in Fig. 3. Regenerating muscles were isolated at day 14 following CTX injection. Representative immunohistochemical images for Laminin α2 in cross-sections of TA muscle (**a**). Cross-sectional area (CSA) of centrally nucleated regenerating myofibers was quantified (**b**), scale bar 50 μm. (*n* = 4). Muscle weight of TA muscles was measured (**c**) (*n* = 3–7). Data represent means ± s.e.m. One-way ANOVA followed by the Bonferroni post hoc test: NS non-significant. **d**–**j** Satellite cell populations were isolated from limb muscles of Pax7^+/+^ or Pax7^YFP/YFP^ mice by FACS and cultured as shown in Fig. 2. Time course for analysis (**d**). **e** Representative images of EdU staining of plated satellite cells in GM conditions [quantified in (**f**)]. Nuclei were stained with DAPI. > 300 cells per mouse were counted (*n* = 3 mice). Data represent means ± s.e.m. (NS non-significant), scale bar 50 μm. **g** Myogenic differentiation was induced under differentiation medium (DM) conditions for 4 days and cells immunostained for MyHC [differentiation index (**h**) was measured as the ratio of MyHC+ nuclei per total DAPI+ nuclei. > 200 cells per mouse were counted (*n* = 6–7 mice)], scale bar 20 μm. Data represent means ± s.d. (NS non-significant). **i**, **j** Self-renewal of satellite cells was induced under DM conditions for 4 days. (**i**) Co-immunostaining for PAX7 (or YFP) and MyoD. Arrows indicate Pax7^+^(or YFP+)/MyoD^−^ self-renewal cells [quantified in (**j**)], scale bar 10 μm. > 250 nuclei per mouse (*n* = 3 mice) were counted. Data represent means ± s.d. (NS non-significant). **k**–**m** To evaluate whether satellite cells isolated from Pax7^YFP/YFP^ mice were able to give rise to progeny and regenerate muscle, transplantation analysis was performed. **k** Time course for analysis of transplantation. YFP^+^ cells (5 × 10^4^ cells) were transplanted into CTX pre-injured TA muscle of mdx mice (*n* = 4). The contralateral legs were injected with PBS and used as a control. Muscles were removed for immunohistochemistry 14 days post-transplantation. **l** Immunohistochemistry for YFP and laminin α2 in cross-sections of TA muscles engrafted with YFP+ satellite cells. Arrows indicate YFP^+^ cells, scale bar 50 μm. **m** Immunohistochemistry of dystrophin and YFP proteins in TA muscle of mdx mice (*n* = 4). Pax7-YFP homozygous mouse-derived satellite cells were capable of producing self-renewed cells and restoring dystrophin in mdx mice. Arrows indicate YFP^+^ cells, scale bar 10 μm

Targeted disruption of *Pax7* in mice leads to a postnatal growth defect and death at 2–3 weeks after birth [23, 25, 27, 29]. In *Pax7*-deficient mice, the number of satellite cells is progressively lost in postnatal stages, and thus, fiber-diameters are significantly reduced [23, 25, 27, 29]. Satellite-cell-specific inactivation of *Pax7* in mice results in a loss of satellite cells, reduced proliferation ability, and precocious myogenic differentiation, thus leading to severe impairment of muscle regeneration [30–32]. In the present study, we examined whether the function of *Pax7* was maintained in satellite cells in Pax7-YFP homozygous (Pax7^YFP/YFP^) mice. Muscle regeneration was induced by CTX injection into the TA muscle of Pax7-YFP knock-in mice, and regenerating muscles were removed 2 weeks after CTX injection. Immunohistochemical analysis showed that the cross-sectional area (CSA) and muscle weight of regenerated muscles from Pax7^YFP/YFP^ mice were both similar to those of Pax7^+/+^ or Pax7^YFP/+^ mice (Fig. 4a–c).

Having shown that muscle regeneration was unlikely to be disturbed by expression of the Pax7-YFP fusion protein, we further determined whether Pax7-YFP KI mouse-derived satellite cells undergo normal myogenic progression in culture ex vivo. The YFP+ satellite cell population was isolated from limb muscles of Pax7^YFP/YFP^ mice and then cultured under GM conditions. Myogenic differentiation was then induced by changing the medium to DM for culturing for 3 days (Fig. 4d). To evaluate proliferation, the EdU pulse-chase assay was performed under GM conditions. The proportion of EdU+ satellite cells from Pax7^YFP/YFP^ mice was not different from that of Pax7^+/+^ mice (Fig. 4e, f). We also confirmed that Pax7^YFP/YFP^ mouse-derived satellite cells undergo myogenic differentiation (Fig. 4g, h) and self-renewal (Fig. 4i, j), similar to Pax7^+/+^ cells. Therefore, our results indicate that the Pax7-YFP fusion protein does not interfere with satellite cell functions, and thus, Pax7^YFP/YFP^ mice efficiently regenerate muscle after injury as well as wild-type mice.

In mdx mice, transplanted satellite cells give rise to progeny in the regenerating niche and reconstitute myofibers [8–13]. We sought to determine whether Pax7-YFP knock-in mouse-derived satellite cells could also be transplanted into limb muscle and regenerate myofibers and self-renew in the host muscle. Satellite cells were isolated from the limb muscles of Pax7^YFP/YFP^ mice by FACS, and 5 × 10^4^ YFP+ cells were grafted into regenerating TA muscle of mdx mice, which had been injected with CTX 1 day prior to the transplantation (Fig. 4k). Non-transplanted muscles were used as a control. Muscles were removed 14 days following transplantation, and transverse sections were immunostained. YFP+ donor-derived satellite cells were detected in the satellite cell niche surrounded by basal lamina (Fig. 4l), and their contribution to muscle regeneration was visualized by dystrophin-expressing myofibers accompanied by YFP+ donor-derived satellite cells (Fig. 4m). These data indicated that Pax7-YFP knock-in mouse-derived satellite cells are transplantable and give rise to newly formed myofibers in regenerating muscles.

## Discussion

The transcription factor PAX7 is an established marker for satellite cells in adult skeletal muscle. Here, we generated a novel knock-in mouse line to enable visualization of PAX7 protein via YFP fluorescence in living satellite cells. Our comprehensive analysis of Pax7-YFP mice demonstrated that YFP fluorescence levels accurately recapitulate endogenous PAX7 protein levels and that all quiescent and undifferentiated satellite cells express YFP protein. YFP+ satellite cells are clearly detectable by immunohistochemistry of cross-sections of both intact and injured muscle tissues in Pax7-YFP knock-in mice, even in the inflammatory stages during muscle regeneration. Also of importance, Pax7^*YFP/YFP*^ homozygous mice are born, grow, and regenerate muscle normally. Satellite cells isolated from Pax7^*YFP/YFP*^ mice proliferate, differentiate, and self-renew as well as those from wild-type Pax7^*+/+*^ mice, indicating that the YFP-tag does not interfere with the function of the endogenous PAX7 protein.

The satellite cell population can be isolated from skeletal muscle tissue of adult mice using satellite-cell-specific cell surface markers (e.g., Vcam1 and α7-integrin) combined with non-myogenic markers (e.g., CD45 and CD31) [11, 12, 41–43]. In the present study, we demonstrated that satellite cells can be highly purified from muscle tissues of Pax7-YFP knock-in mice by FACS without antibody staining, similar to cells isolated from transgenic *Pax7-ZsGreen*, *Pax7-nGFP*, and *Pax7-GFP* reporter mouse lines that have recently been reported [34–36]. Recently, Rocheteau et al. reported that a *Pax7*^high^ cell population retains stemness in quiescent satellite cells using *Pax7-nGFP* reporter adult mice [35]. Our Pax7-YFP knock-in mouse line could be applicable to further characterize the stem-like population. FACS isolated YFP+ satellite cells from our mouse line are also transplantable into regenerating muscle of mdx mice and give rise to progeny as well as newly formed myofibers. Indeed, we believe Pax7-YFP mice will be useful for developing stem-cell-based therapies for muscle diseases.

## Conclusions

We established a Pax7-YFP knock-in mouse line to further understand the function and dynamics of PAX7 protein in satellite cells. This knock-in mouse line is applicable for in vitro and in vivo live cell-imaging analysis of satellite cell dynamics via YFP fluorescence. Because Pax7-YFP mouse-derived satellite cells express YFP-tagged PAX7 protein, they can also be used for ChIP-seq analysis to identify PAX7-regulated genes. Recent studies have described the functions of *Pax7* in muscle diseases: PAX7-target genes are globally repressed by the DUX4 transcription factor, which is ectopically expressed in muscles of facioscapulohumeral muscular dystrophy (FSHD) patients [45]. Furthermore, in a mouse model of cancer cachexia, PAX7 protein is highly upregulated, which suppresses myogenic differentiation of satellite cells, leading to muscle atrophy [46]. Therefore, we hope that our Pax7-YFP mouse line will facilitate investigation of satellite cell biology and will benefit the development of stem-cell-based therapies to treat muscle diseases.

## Additional file

Additional file 1: **Table S1.** Primers for quantitative PCR. (PDF 22 kb)

## Abbreviations

CSA: Cross-sectional area
CTX: Cardiotoxin
DM: Differentiation medium
DMD: Duchenne muscular dystrophy
EDL: Extensor digitorum longus
FACS: Fluorescence-activated cell sorting
FSHD: Facioscapulohumeral muscular dystrophy
GM: Growth medium
PFA: Paraformaldehyde
Q-PCR: Quantitative revere transcription-PCR
Sol: Soleus
TA: Tibialis anterior
YFP: Yellow fluorescent protein
