# Supplementary material for: Visualization of PAX7 protein dynamics in muscle satellite cells in a YFP knock-in-mouse line
Source: Skelet Muscle. 2018 Aug 24;8:26. doi: 10.1186/s13395-018-0174-x (PMC6108100; doi:10.1186/s13395-018-0174-x)
Supplement: Supplementary file 1 — Table S1. Primers for quantitative PCR. (PDF 22 kb) [file 13395_2018_174_MOESM1_ESM.pdf]

Pax7-F	GTGCCCTCAGTGAGTTCGATTAGC	Pax7-R	CCACATCTGAGCCCTCATCCA
YFP-F	CCTGTGGAAACGGGACAAGC	YFP-R	CGGACACGCTGAACTTGTGG
Myf5-F	TGAGGGAACAGGTGGAGAAC	Myf5-R	AGCTGGACACGGAGCTTTTA
MyoD-F	AGCACTACAGTGGCGACTCA	MyoD-R	GCTCCACTATGCTGGACAGG
Myogenin-F	CTACAGGCCTTGCTCAGCTC	Myogenin-R	AGATTGTGGGCGTCTGTAGG
GAPDH-F	AACTTTGGCATTGTGGAAGG	GAPDH-R	CACATTGGGGGTAGGAACAC
Myh3-F	GCCAGGATGGGAAAGTCACTGTGG	Myh3-R	GGGCTCGTTCAGGTGGGTCAGC

Supplementary Table 1
